# Activity-Dependent Synaptic Refinement: New Insights from *Drosophila*

**DOI:** 10.3389/fnsys.2017.00023

**Published:** 2017-04-21

**Authors:** Fernando Vonhoff, Haig Keshishian

**Affiliations:** Department of Molecular, Cellular and Developmental Biology, Yale UniversityNew Haven, CT, USA

**Keywords:** oscillation, chemorepulsion, neuromuscular junction, non-Hebbian, second messengers

## Abstract

During development, neurons establish inappropriate connections as they seek out their synaptic partners, resulting in supernumerary synapses that must be pruned away. The removal of miswired synapses usually involves electrical activity, often through a Hebbian spike-timing mechanism. A novel form of activity-dependent refinement is used by *Drosophila* that may be non-Hebbian, and is critical for generating the precise connectivity observed in that system. In *Drosophila*, motoneurons use both glutamate and the biogenic amine octopamine for neurotransmission, and the muscle fibers receive multiple synaptic inputs. Motoneuron growth cones respond in a time-regulated fashion to multiple chemotropic signals arising from their postsynaptic partners. Central to this mechanism is a very low frequency (<0.03 Hz) oscillation of presynaptic cytoplasmic calcium, that regulates and coordinates the action of multiple downstream effectors involved in the withdrawal from off-target contacts. Low frequency calcium oscillations are widely observed in developing neural circuits in mammals, and have been shown to be critical for normal connectivity in a variety of neural systems. In *Drosophila* these mechanisms allow the growth cone to sample widely among possible synaptic partners, evaluate opponent chemotropic signals, and withdraw from off-target contacts. It is possible that the underlying molecular mechanisms are conserved widely among invertebrates and vertebrates.

It is estimated that the nearly 10^11^ neurons of the human nervous system establish over 10^14^ synaptic connections (Azevedo et al., 2009; Kasthuri et al., 2015). To wire up a system of such astonishing complexity requires mechanisms that are highly efficient and flexible. Rather than uniquely specifying each synaptic connection, the developing nervous system can initially establish connections that are characterized by supernumerary synaptic contacts, as widely observed in neural networks. Inappropriate off-target synapses are subsequently pruned away through activity-dependent mechanisms to yield a more precise and functional connectome (reviewed in Katz and Shatz, 1996; Yamamoto and López-Bendito, 2012; Doll and Broadie, 2014; Koropouli and Kolodkin, 2014; Arroyo and Feller, 2016). Errors in synaptic pruning are associated with several neurological disorders, including autism and schizophrenia (Berridge, 2012; Tang et al., 2014; Sekar et al., 2016).

In this review article, we examine synaptic refinement with a focus on the embryonic and larval neuromuscular system of *Drosophila*, where some of the underlying molecular mechanisms have been resolved (Carrillo et al., 2010; Vonhoff and Keshishian, 2017). This simple array of synapses is established by two distinct classes of motoneurons that use as neurotransmitters either glutamate (Johansen et al., 1989) or the biogenic amine octopamine (Monastirioti, 1999).

## Activity Dependent Refinement

The refinement of neural connections occurs in vertebrates and invertebrates, and has been extensively studied in the developing visual system (reviewed in D’Orazi et al., 2014; Pratt et al., 2016). Although activity-independent synapse elimination has been observed in mouse retinal cells (Morgan et al., 2011; Wei et al., 2011; Yonehara et al., 2011), activity-dependent mechanisms play a crucial role in establishing precise network connectivity (reviewed in Huberman et al., 2008; Cang and Feldheim, 2013). Pioneering work by Hubel and Wiesel showed that visual experience was required for the formation of ocular dominance columns between axons of the lateral geniculate nucleus (LGN) of the thalamus, and layer 4 neurons in primary visual cortex (Wiesel and Hubel, 1963). The requirement for neural activity in the segregation of visual projections was subsequently tested using TTX eye injections in both cold blooded vertebrates (Meyer, 1982), and in mammals (Shatz and Stryker, 1988; Sretavan et al., 1988). Patterned neural activity was also found to be essential for refining retinotopic map projections at other visual centers, such as the superior colliculus (McLaughlin et al., 2003). Activity-dependent refinement is also involved in controlling the balance between excitatory and inhibitory synapses, as found for the Xenopus optic tectum (Akerman and Cline, 2007). Elsewhere activity is involved in the elimination of supernumerary contacts at the vertebrate neuromuscular junction (reviewed by Sanes and Lichtman, 2001), and for synapse elimination of climbing fiber inputs to cerebellar Purkinje cells (reviewed by Purves and Lichtman, 1980; Kano and Hashimoto, 2009).

The remodeling that occurs during synaptic refinement suggests that electrical activity influences neurite growth or retraction. The link between activity and growth is a general feature of neural systems. For example, in *Drosophila* altered levels of neural activity in embryonic olfactory projection neurons (Prieto-Godino et al., 2012) and in larval and adult motoneurons (Duch et al., 2008; Hartwig et al., 2008) affects dendrite size and complexity, and thus directly influences synaptic connections. Similarly, in larval motoneurons manipulation of neural activity alters presynaptic NMJ size and arbor complexity, and affects presynaptic bouton morphology (Budnik et al., 1990; Zhong et al., 1992; Lnenicka et al., 2003; Mosca et al., 2005; Berke et al., 2013).

## Molecular Mechanisms Underlying Refinement

How is neural activity linked to the cell biology of neuronal growth and retraction? Depolarization elevates intracellular free calcium (Ca^2+^) levels through voltage-gated calcium channels (VGCCs). As a result, the mechanisms regulating synaptic connectivity generally involve Ca^2+^-dependent effectors. Ca^2+^-dependent signaling can influence early growth events, such as the motility and exploration of the growth cone (Kater and Shibata, 1994; Zheng and Poo, 2007; Rosenberg and Spitzer, 2011). In some cases this is due to the modulation of the growth cone’s response to various exogenous chemotropic factors, such as netrin-1-induced attraction, myelin-associated glycoprotein (MAG)-induced repulsion (Ming et al., 2001), or Ephrin-A induced repulsion of mouse retinal ganglion cells (Nicol et al., 2007).

Within the cytoplasm, Ca^2+^ regulates the activity of various GTPases (Jin et al., 2005), that in turn affect cytoskeletal dynamics within the growing contact. GTPases serve as a key molecular link between changes in free Ca^2+^ levels in the growth cone due to activity, and subsequent responses to chemotropic factors (Lowery and Van Vactor, 2009). One potential mechanism linking neural activity and cytoskeletal dynamics would involve the regulation of actin by the activity of Rho GTPases. Rho is known to regulate ROCK function, which in turn activates LIM Kinase (LIMK; Amano et al., 2010). LIMK inhibits cofilin, an actin severing protein that promotes actin recycling. Consistent with this hypothesis, LIMK is known to regulate synaptic function in mice (Meng et al., 2002) as well as NMJ growth in *Drosophila* (Ang et al., 2006).

A second molecular mechanism regulating activity-dependent refinement involves interactions between Ca^2+^ and cyclic nucleotides such as cAMP and cGMP. Intracellular cyclic nucleotide levels regulate chemotropic growth cone turning (Lohof et al., 1992; Song et al., 1997; Nishiyama et al., 2003), synaptic plasticity (Zhong et al., 1992), and the refinement of axon branches in both retinal cells (Nicol et al., 2006) and *Drosophila* motoneurons (Vonhoff and Keshishian, 2017). Whether cAMP levels are positioned upstream or downstream of Ca^2+^ signaling remains incompletely resolved, as there is evidence in the literature for both scenarios. cAMP levels may act downstream of Ca^2+^ as connectivity defects arise following misregulation of Ca^2+^-dependent adenylyl cyclases, such as AC1 in mouse retinal neurons (Nicol et al., 2006), ADCY8 in zebrafish retinal neurons (Xu et al., 2010), and Rutabaga in *Drosophila* motoneurons (Vonhoff and Keshishian, 2017). By contrast, cAMP also regulates Ca^2+^-signaling as it promotes Ca^2+^-induced Ca^2+^-release (CICR) from internal stores (Gomez and Zheng, 2006; Zheng and Poo, 2007), modulates the amplitude of growth cone Ca^2+^-transients (Nicol et al., 2011), and cyclic nucleotide-gated (CNG) ion channels, to allow for Ca^2+^-influx in growth cones (Togashi et al., 2008).

Intracellular Ca^2+^ activates several pathways that converge on transcription factors that control the expression of activity-regulated genes that may be involved in guidance mechanisms. This was first revealed for the immediate early gene c-fos, downstream of Ca^2+^ influx (Greenberg et al., 1986). Fos protein together with Jun family members comprises the AP-1 transcription factor (Curran and Franza, 1988). AP-1 has been involved in synaptic plasticity in mouse hippocampal neurons (Fleischmann et al., 2003) as well as in activity-dependent dendritic growth of *Drosophila* motoneurons (Hartwig et al., 2008; Vonhoff et al., 2013) and synaptic development at the *Drosophila* NMJ (Sanyal et al., 2002).

Finally, there is good evidence from both vertebrates and invertebrates that synaptic refinement requires temporally patterned changes or oscillations in the levels of second messengers. This dynamism has been particularly evident for Ca^2+^, where spontaneous retinal waves are critical for the refinement of visual maps in the mouse brain (Wong, 1999; Arroyo and Feller, 2016), as well as for the refinement of neuromuscular junctions in *Drosophila* embryos (Carrillo et al., 2010; Vonhoff and Keshishian, 2017). It is intriguing that cAMP levels are also required to oscillate for the refinement of mouse retinal axons (Nicol et al., 2007), or to be dynamically maintained within an optimal level for the refinement of *Drosophila* motoneuron axon branches (Vonhoff and Keshishian, 2017).

## *Drosophila* NMJ as a Genetic Model to Study Synaptic Refinement

The *Drosophila* larval bodywall offers an anatomically stereotypic genetic model system for studying many aspects of neuronal connectivity (for reviews see Ruiz-Cañada and Budnik, 2006; Menon et al., 2013). Among its features are singly identifiable glutamatergic motoneurons with very narrow connectivity, innervating only one or two muscle fibers each, and a subset of efferent neuromodulatory neurons that express the biogenic amine octopamine (Monastirioti et al., 1995, 1996; Monastirioti, 1999) that project widely and innervate multiple muscle fibers.

The stereotypic connectivity of the embryonic and larval *Drosophila* NMJ crucially relies on the expression of molecular recognition cues (reviewed in Nose, 2012). Whereas some molecules are expressed by all muscles, the expression pattern of other cues is restricted to individual muscles (Winberg et al., 1998). Examples of muscle-specific cues include Fasciclin III (Halpern et al., 1991), Capricious (Shishido et al., 1998), Connectin (Nose et al., 1992), and NetrinB (Harris et al., 1996). By contrast, other molecules are expressed by numerous muscle fibers, as for example Fasciclin II (Lin and Goodman, 1994), Teneurin-m (Mosca et al., 2012), Dpr11 (Carrillo et al., 2015), and Semaphorin2a (Matthes et al., 1995).

**Figure 1:**
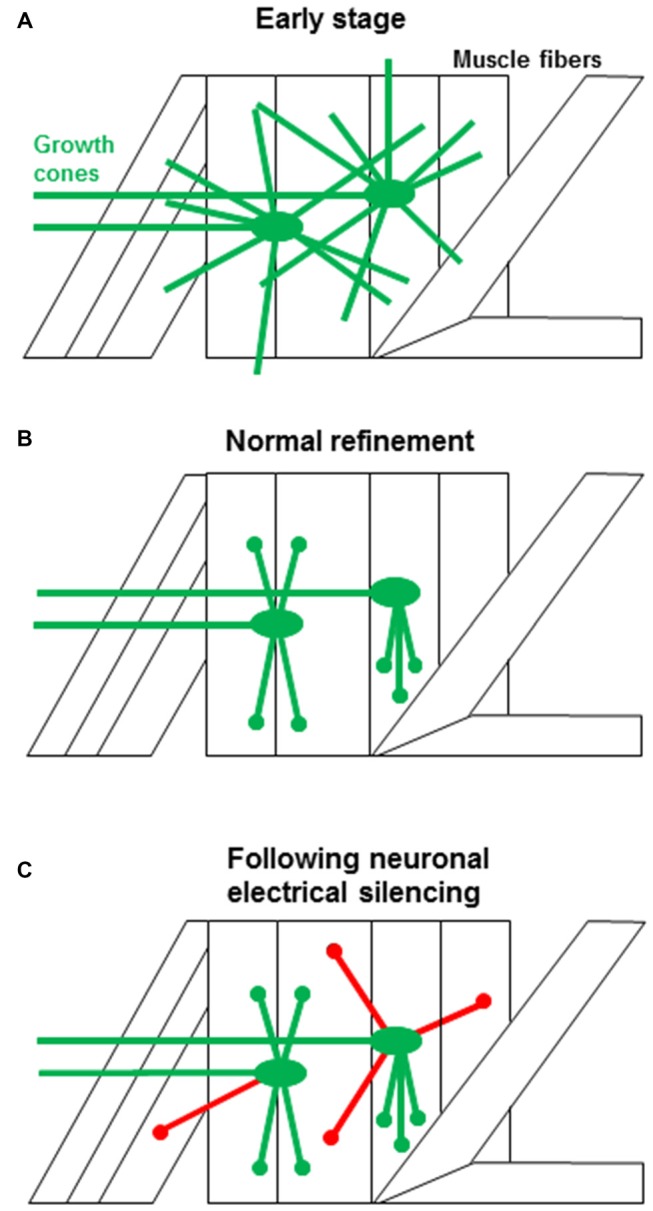
**The events associated with synaptic targeting at the *Drosophila* NMJ. (A)** Initial motoneuron projections make filopodial contacts (green) onto both the target muscle as well as to multiple off-target muscle fibers (Halpern et al., 1991; Sink and Whitington, 1991). **(B)** During normal development, off-target contacts are withdrawn, leading to the final specific connectivity (green). The refinement must occur during an early critical period and depends on presynaptic electrical activity (Jarecki and Keshishian, 1995; White et al., 2001; Carrillo et al., 2010). **(C)** When neural activity is suppressed, the off-target contacts are retained (red), leading to ectopic synapses. The transition from a growth cone filopodium to a synapse is rapid and the refinement of ectopic contacts occurs while growth cones are still motile, consistent with the critical period for refinement at the *Drosophila* NMJ. Ectopic contacts that fail to withdraw develop into functional synapses. This is in contrast to scenarios observed in other systems where synaptic contacts have to be stabilized and then refined by mechanisms that rely on prolonged period of synaptic competition.

During embryonic development *Drosophila* motoneuron growth cones sample widely among muscle fibers, and inevitably make inappropriate contacts, as shown schematically in Figure 1A (Halpern et al., 1991; Sink and Whitington, 1991; Chiba et al., 1993). The off-target contacts are removed during an early critical period (late embryo to early 1st instar; Figure 1B), otherwise they mature into functional ectopic synapses (Jarecki and Keshishian, 1995; Carrillo et al., 2010). Ultimately, neural activity refines the motoneuron contacts, so that their connectivity is limited only to their appropriate muscle fiber targets. Silencing electrical activity in the motoneurons during the critical period increases the frequency of ectopic motoneuron contacts throughout the bodywall (Figure 1C; Jarecki and Keshishian, 1995; White et al., 2001; Carrillo et al., 2010).

*In vivo* electrical activity in the embryo is highly patterned, with brief (~15 s) bursts of action potentials spaced every 2–3 min (Pereanu et al., 2007; Crisp et al., 2008; Vonhoff and Keshishian, 2017). Normal synaptic refinement depends on the presence of two voltage-gated Ca^2+^ channels, Cacophony (Cac), the Ca(v)2.1 channel (Carrillo et al., 2010), and Dmca1G, the Ca(v)3 channel (Vonhoff and Keshishian, in preparation). The experimental rescue of the *cac* mutation to restore normal synaptic connectivity requires oscillatory presynaptic Ca^2+^ entry, timed to resemble the native electrical oscillations (Carrillo et al., 2010). This indicates that Ca^2+^-oscillations at a specific frequency and pattern (in the range of 0.01–0.03 Hz) are required for proper synaptic refinement.

**Figure 2:**
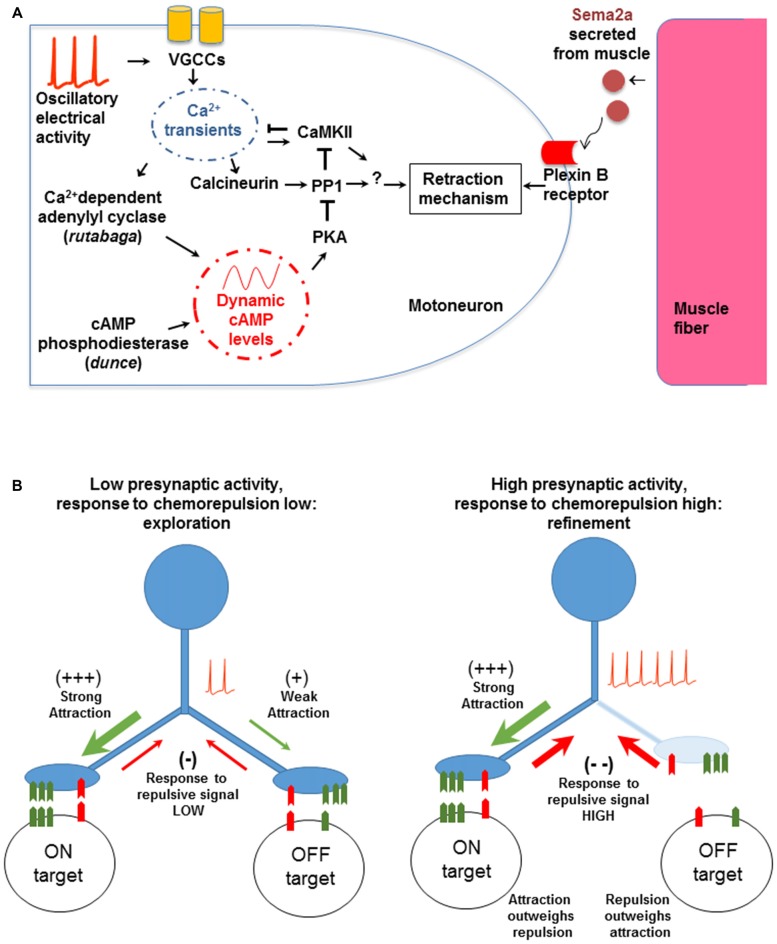
**The molecular and cellular mechanisms involved in synaptic refinement. (A)** The interactions were identified by genetic tests and transgenic manipulations. A low frequency voltage oscillation activates voltage gated Ca^2+^ channels (VGCCs). The resulting Ca^2+^ entry regulates Ca^2+^-dependent effectors including Ca^2+^/calmodulin-dependent serine/threonine kinase II (CaMKII), Calcineurin (CaN), and Rutabaga. The latter increases cAMP levels, which in turn regulate PKA and PP1. The chemorepellant Sema2a is secreted by the muscle and activates the presynaptic PlexinB receptor. The response to Sema2a is gated by the level of presynaptic Ca^2+^ activity (see text for details). Arrows and T-shape lines indicate positive and negative regulation, respectively. The subcellular physical location and region of action of the molecular components have not been determined yet. **(B)** A model for non-Hebbian refinement at the *Drosophila* NMJ. The left panel shows an initial contact made by a motoneuron onto on-target and off-target muscle fibers. The molecular match is stronger with the on-target fiber. When Ca^2+^ levels are low, the response to the retrograde chemorepulsive signal from the muscle is muted, allowing the off-target contact to be retained. With neural activity and elevated presynaptic Ca^2+^ (right panel), the repulsive response is elevated, leading to the withdrawal of the off-target contact. Note that the model does not depend on correlated activity between the synaptic partners, as would be expected in a Hebbian mechanism.

In addition to the activity-dependent entry of Ca^2+^ through Ca^2+^ channels (Figure 2A), refinement also depends on the activity of at least three downstream Ca^2+^-dependent signaling systems in the presynaptic terminal: the Ca^2+^/calmodulin-dependent serine/threonine kinase II (CaMKII; Carrillo et al., 2010), the Ca^2+^/calmodulin-dependent serine/threonine protein phosphatase Calcineurin (CaN; Vonhoff and Keshishian, in preparation), and the Ca^2+^-dependent adenylyl cyclase Rutabaga (Vonhoff and Keshishian, 2017). Rutabaga elevates intracellular cAMP-levels, which are degraded by the activity of the cAMP-phosphodiesterase Dunce. Similarly, molecules whose activity is typically downstream of cAMP such as PKA and PP1 are also required for synaptic refinement (Vonhoff and Keshishian, 2017). Notably, PKA and CaN are known to interact with PP1 (Blitzer et al., 1998; Oliver and Shenolikar, 1998), which in turn can regulate CaMKII. Collectively, these interactions suggest a complex signaling network to govern synaptic refinement in this system (Figure 2A).

How are off-target contacts withdrawn? There is strong evidence that synaptic pruning depends on an active response by the presynaptic growth cone to Sema2a, a chemorepulsive molecule secreted by muscle fibers that acts via the PlexinB receptor in motoneurons (Winberg et al., 1998; Ayoob et al., 2006; Carrillo et al., 2010). We hypothesize that Ca^2+^ entry into the developing motoneuron terminal modulates the cell’s chemorepulsive response to Sema2a. A similar role for neural activity and Ca^2+^ waves in modulating chemotropic and guidance responses of growth cones has been proposed for vertebrate neurons (Spitzer et al., 2000; Ming et al., 2001; Nicol et al., 2006, 2011; Rosenberg and Spitzer, 2011).

We therefore propose a model where the response of the motoneuron growth cone to muscle-derived Sema2a is episodically modulated in an oscillatory fashion (Figure 2B). When Ca^2+^-levels in growth cones are low, exploratory filopodia are favored to contact and extend on membrane surfaces. By contrast, during activity bouts, Ca^2+^- and cAMP levels transiently increase, raising the responsiveness of the neuron to the Sema2a-chemorepellant and withdrawing the less firmly-associated filopodial contacts from off-target surfaces. Thus, presynaptic electrical activity regulates complex molecular interactions in a time-dependent fashion, to modulate the neuron’s responsiveness to chemorepulsion exerted by the muscle fibers. These results provide a coherent picture of the links between neural activity, chemorepulsion, and the refinement of synaptic connectivity.

## Molecular Candidates that may be Involved in Activity-Dependent Refinement

Although a crucial role for Ca^2+^-influx via VGCCs in the withdrawal of off-target neuromuscular contacts has been observed, a role for CICR in synaptic refinement in *Drosophila* remains untested. CICR is influenced by cAMP (Gomez and Zheng, 2006; Zheng and Poo, 2007) and is required for netrin-1 induced growth cone turning (Hong et al., 2000). Furthermore, filopodial Ca^2+^ transients have been shown to activate the protease calpain to promote growth cone repulsive turning (Robles et al., 2003). Several calpain genes with neural expression have been identified in *Drosophila* (Friedrich et al., 2004), and have been associated with Ca^2+^-dependent dendrite pruning (Kanamori et al., 2013), offering a potential regulatory mechanism for future examination.

Alternative links between neural activity and CaN for synaptic refinement also remain untested, as for example molecular pathways involving the activity-dependent transcription factor AP1. In murine T-cells, CaN dephosphorylates NFAT, a DNA-binding phosphoprotein that forms a complex with Fos and Jun to activate gene transcription (Jain et al., 1993). In cultured mouse primary neurons, the CaN-NFAT signaling is required to promote the netrin-1 dependent axonal outgrowth (Graef et al., 2003). In *Drosophila* motoneurons, AP1 promotes activity-dependent dendritic growth (Hartwig et al., 2008; Vonhoff et al., 2013) and synaptic plasticity (Sanyal et al., 2002) together with NFAT at the larval NMJ (Freeman et al., 2011). Furthermore, CaN and the GSK-3β kinase homolog Shaggy have been recently described to regulate bouton stabilization at the larval NMJ by activating or inhibiting the microtubule associated protein-1b fly ortholog futsch/MAP-1b, respectively (Wong et al., 2014). Shaggy activates the CaN-regulator Sra in *Drosophila* eggs (Takeo et al., 2012), and also negatively regulates neuronal AP1 function by inhibiting the JNK pathway, as described in an *in vivo* genetic screen in *Drosophila* (Franciscovich et al., 2008). Interestingly, the genes *sema2a* and *fkbp13* (a protein predicted to bind the pharmacological agent FK506, a known inhibitor of CaN) were identified in the same screen among the molecules that regulate AP1 function (Franciscovich et al., 2008). Whether these genes play a role in the activity-dependent withdrawal of ectopic contacts or in the modulation of chemorepulsion remains to be tested.

## Biogenic Amines and Refinement

Synaptic connectivity in *Drosophila* can range from precise targeting, as seen for the glutamatergic motoneurons that limit their connections to just one or two bodywall muscle fibers, to efferents that establish broad projections across the musculature, such as those expressing the biogenic amine octopamine. To what extent are the molecular mechanisms governing guidance and synaptic refinement conserved between these two distinct patterns of synaptic connectivity?

The octopaminergic motoneurons are highly plastic and respond to elevated electrical activity by expanding their peripheral arbors on the musculature (Zhong et al., 1992; Budnik, 1996; Koon et al., 2011). Although the octopaminergic projections are made over a broad expanse of the musculature, the wiring is nevertheless subject to activity-dependent refinement. Over half of the activity-dependent ectopic contacts found on muscle fibers are made by the octopaminergic motoneurons and those ectopic contacts are largely eliminated when neuromuscular activity is normal (Jarecki and Keshishian, 1995; Carrillo et al., 2010; Vonhoff and Keshishian, 2017). Thus similar mechanisms are likely at play to refine the connections made by the glutamatergic motoneurons that project to only one or two muscle fibers and the octopaminergic neurons that project to large regions of the musculature.

Octopamine regulates the activity-dependent plasticity of glutamatergic motoneurons in a paracrine fashion, acting through Octβ2R receptors that regulate cAMP levels at the NMJs (Koon et al., 2011; Koon and Budnik, 2012). It is therefore possible that the octopaminergic efferents are themselves involved in regulating synaptic refinement. *Drosophila* expresses four distinct octopamine receptors (El-Kholy et al., 2015), including multiple forms that are found in neurons and muscles. As the *Drosophila* octopamine GPCRs modulate cAMP levels as well as Ca^2+^ signaling (Balfanz et al., 2005; Evans and Maqueira, 2005; Maqueira et al., 2005; Maiellaro et al., 2016), this raises the possibility that octopamine influences the refinement process by modulating the levels of these second messengers.

## Concluding Thoughts

The refinement of synaptic connections often involves Hebbian, spike-timing correlation between synaptic partners, with asynchronous inputs removed (an idea first elaborated by Stent, 1973). This ubiquitous mechanism is involved in topographic map development and synaptic refinement throughout the vertebrate CNS. By contrast, the *Drosophila* NMJ apparently does not require postsynaptic depolarization for the removal of off-target contacts (Jarecki and Keshishian, 1995; White et al., 2001; Carrillo et al., 2010), suggesting a fundamentally different mechanism for synaptic refinement. Moreover, there is no evidence for competition based on correlated synaptic activity at the *Drosophila* NMJ, as is the case for refinement in other systems.

At the *Drosophila* NMJ connectivity is governed by a combinatorial system of recognition molecules expressed by motoneurons and muscles. A correct molecular “match” is needed to stabilize the motoneuronal contact leading to a functional synapse (Furrer and Chiba, 2004; Menon et al., 2013; Carrillo et al., 2015). As noted above, the motoneurons sample among possible synaptic partners, with off-target contacts withdrawn in an activity-dependent fashion. The challenge is to make guidance decisions based on opponent signals that are presented simultaneously: a global chemorepellant signal from all muscles, and a local chemoattractive signal from the target cell. Assuming that the response to the chemorepellant is governed by Ca^2+^ levels, then the growth cone sampling and withdrawal phases would be coordinated by the Ca^2+^ oscillations (Figure 2B). We view this mode of error correction as a form of time-dependent signal multiplexing, where the neuron can respond to distinct chemotropic signals depending on the phase of the Ca^2+^ oscillation. Vital imaging experiments currently underway (Vonhoff and Keshishian, in preparation), are testing whether there is a direct correlation between growth cone motility and the underlying low frequency Ca^2+^ oscillation.

## Author Contributions

FV and HK wrote the manuscript and designed the figures.

## Funding

This study was supported by the National Institutes of Health (grant no. 1R21NS053807, 5R01NS031651).

## Conflict of Interest Statement

The authors declare that the research was conducted in the absence of any commercial or financial relationships that could be construed as a potential conflict of interest.
